# Long term outcome of functional independence and quality of life after traumatic SCI in Germany

**DOI:** 10.1038/s41393-021-00659-9

**Published:** 2021-06-25

**Authors:** Florian Möller, Rüdiger Rupp, Norbert Weidner, Christoph Gutenbrunner, Yorck B. Kalke, Rainer F. Abel

**Affiliations:** 1 Spinal Cord Injury Center, Heidelberg University Hospital, Heidelberg, Germany; 2 Department of Rehabilitation Medicine, Hannover Medical School, Hanover, Germany; 3 Orthopedic Department, SCI Centre - Ulm University, Ulm, Germany; 4 Klinik Hohe Warte, Hospital Bayreuth, Bayreuth, Germany

**Keywords:** Trauma, Spinal cord diseases

## Abstract

**Study design:**

Multicenter observational study.

**Objective:**

To describe the long-term outcome of functional independence and quality of life (QoL) for individuals with traumatic and ischemic SCI beyond the first year after injury.

**Setting:**

A multicenter study in Germany.

**Methods:**

Participants of the European multicenter study about spinal cord injury (EMSCI) of three German SCI centers were included and followed over time by the German spinal cord injury cohort study (GerSCI). Individuals’ most recent spinal cord independence measure (SCIM) scores assessed by a clinician were followed up by a self-report (SCIM-SR) and correlated to selected items of the WHO short survey of quality of life (WHO-QoL-BREF).

**Results:**

Data for 359 individuals were obtained. The average time passed the last clinical SCIM examination was 81.47 (SD 51.70) months. In total, 187 of the 359 received questionnaires contained a completely evaluable SCIM-SR. SCIM scores remained stable with the exception of reported management of bladder and bowel resulting in a slight decrease of SCIM-SR of −2.45 points (SD 16.81). SCIM-SR scores showed a significant correlation with the selected items of the WHO-QoL-BREF (*p* < 0.01) with moderate to strong influence.

**Conclusion:**

SCIM score stability over time suggests a successful transfer of acquired independence skills obtained during primary rehabilitation into the community setting paralleled by positively related QoL measurements but bladder and bowel management may need special attention.

## Introduction

Achieving the highest level of functional independence is one of the main objectives of primary rehabilitation of individuals with SCI.

Former studies were able to show a favorable relationship between functional independence at discharge and multiple long-time outcomes such as rehospitalization rates, probability of living in a community setting, and employment status. There is a wealth of data analyzing the course of functional independence within the first year after the onset of SCI and the relationship with different aspects of quality of life (QoL) for individuals living with SCI. However, data following individuals’ independence and correlation with QoL over a long-time period are rare [1–3].

In 2013, with their initiative “International perspectives on spinal cord injury” the World Health Organization (WHO) invited to investigate the “lived experience of people with SCI across the life course and throughout the world” [4]. In response to the request of the WHO a cooperative effort of two major German scientific societies with a strong focus on rehabilitation of individuals with SCI (German Medical SCI society (DMGP) and German Society for Physical Medicine and Rehabilitation (DGPRM)) started the project “German Spinal Cord Injury Cohort Study” (GerSCI) as part of the “International Spinal Cord Injury Survey” (InSCI).

### Objectives

Within the GerSCI project, we describe and analyze individuals’ long-term changes of functional independence in a community-dwelled setting. We hypothesize that (1) an overall stable course of these variables can be observed, and (2) individuals’ reported QoL is positively related to their level of functional independence.

## Methods

### Study design

Multicenter observational study linking clinical data of individuals with traumatic or ischemic SCI obtained in the first year after the onset of SCI as part of the European multicenter study about spinal cord injury (EMSCI) to long-term data derived from a longitudinal study (GerSCI) on self-reported independence and QoL.

### Participants and collection of data

The participants of our survey were followed within the context of the GerSCI study. GerSCI inclusion criteria are at least one SCI-related hospital admission (initial admission as well as any other in the course of time) at a minimum age of 18 years in the participating centers between January 1995 and December 2016, sufficient knowledge of German language, domestic residency, completed initial rehabilitation and SCI onset at least 12 months ago. GerSCI excluding criteria as congenital and neurodegenerative SCI correspond with the excluding criteria of EMSCI [5, 6].

For three EMSCI centers (Bayreuth, Heidelberg, and Ulm) participants were systemically screened for possible participation in the GerSCI study.

The dataset of the EMSCI examination contains results from five defined points (<15 days, 1, 3, 6, and 12 months) after SCI onset. The results of the latest available examination and contact details were extracted from the EMSCI database. Individuals were designated with a GerSCI ID and the EMSCI ID was deleted from the dataset. The GerSCI questionnaire was mailed to the EMSCI participants in April 2017 (Ulm), July 2017 (Bayreuth), and November 2017 (Heidelberg) followed by a reminder in case no response has been received within 4 weeks.

After mailing the reminder remaining individual-related data (e.g., contact details) were deleted from our dataset which was thereby anonymized. The subsequent individual allocation of GerSCI results to the dataset solely was performed with the help of individuals GerSCI ID.

All GerSCI responses were individually mailed by the study participants to the GerSCI coordinating site, the Department for Rehabilitation Medicine of the Hannover Medical School (Prof. Dr. Gutenbrunner), and entered into a database using dedicated software (Weingabe; Rolf Rimmele, Altenholz, Germany) by trained staff members. Data input was performed twice and any deviations were retraced and cleared by a senior staff member. Alternatively, individuals could answer an online questionnaire. When using the paper-pencil questionnaire data was checked for incoherent responses to connected items with conditional response options. Finally, individuals GerSCI results were connected to the dataset.

### Outcome measures

The spinal cord independence measure (SCIM) is an established outcome measure for functional independence. It was developed to account for the specific aspects of measuring the functional independence of individuals with SCI. Its third version (SCIM-III), developed from the initial version introduced in 1997, has gained large acceptance in the SCI community as a reliable assessment of functional impairments [7]. The SCIM-III consists of 19 items organized in the three sub-scales “self-care”, “respiration and sphincter management” and “mobility” [8]. The EMSCI database is inter alia including International Standards of Neurological Classification of Spinal Cord Injury (ISNCSCI) and SCIM-III assessments at defined time points during the first year after injury, representing the largest collection of SCIM assessments ever recorded. The SCIM-III is available in a validated observation-based, interview-based, and self-reported (SCIM-III-SR) version. The latter was introduced into the GerSCI survey. Even if possibly biased by a home-dwelled setting and unsupervised self-reporting, former studies have found the self-reported version to be comparable to the interviewed and observed versions [7–9]. Differences of participants’ sum-score and sub-scores between the last SCIM-III examination within the EMSCI study and the SCIM-III-SR surveyed within GerSCI were analyzed. Whenever possible (fully completed SCIM-III-SR within GerSCI) differences of the sum-scores were analyzed. In addition, differences of all sub-scores were analyzed.

The questionnaire of the GerSCI study also contained six selected items of the short survey of quality of life by the WHO (WHO-QoL-BREF) [10]. Included items address the perceived overall life quality (WHO-QoL-BREF item 1), overall health status (item 2), ability to perform daily living activities (item 17), self-satisfaction (item 19), satisfaction with personal relationships (item 20), and the satisfaction with the conditions of the personal living place (item 23) reported on a five-tier Likert-scale [11]. Since GerSCI only included six items of this inventory the analysis performed was different from the standardized evaluation as defined by the WHO. As target measurement for QoL item 1 (“How would you rate your quality of life?”) addressing the overall life quality was chosen. As target measurement for independence item 17 (“How satisfied are you with your ability to take care of everyday tasks?”) addressing the ability to perform daily living activities was selected.

### Statistics

Data analysis was carried out using “Statistical Package for the Social Sciences (SPSS)” version 22 (International Business Machines (IBM) Corporation, Armonk, NY, USA).

Descriptive statistics present the sample characteristics of responding and non-responding individuals as well as of evaluability of the returned SCIM-III-SR questionnaires. In addition, the connection between frequently occurring incoherent responses to SCIM-III-SR and level of lesion (tetra-/paraplegia) (Mann–Whitney–U-testing), age (regression analysis), and level of formal education (correlation analysis) was investigated [12]. Linear regression was used to determine a relationship between differences of SCIM sum-scores and sub-scores and time since the last EMSCI examination. Paired *t*-testing was used to analyze differences between SCIM sum-scores and sub-scores obtained in EMSCI and at follow-up in GerSCI.

Scores to items of the WHO-QoL-BREF are presented by descriptive statistics, the connection between these scores and the level of lesion (tetra-/paraplegia) was analyzed by Kruskal–Wallis-testing, Spearman’s rank correlation analysis was performed to investigate the dependency of QoL on individuals’ SCIM-III-SR sum scores and sub-scores.

Possible center effects were investigated for key demographic characteristics (responding and non-responding individuals), differences in SCIM sub-scores and sum-score over time (responding individuals), and for QoL measurements (responding individuals) by Kruskal–Wallis-testing.

## Results

### Sample characteristics

**Fig. 1:**
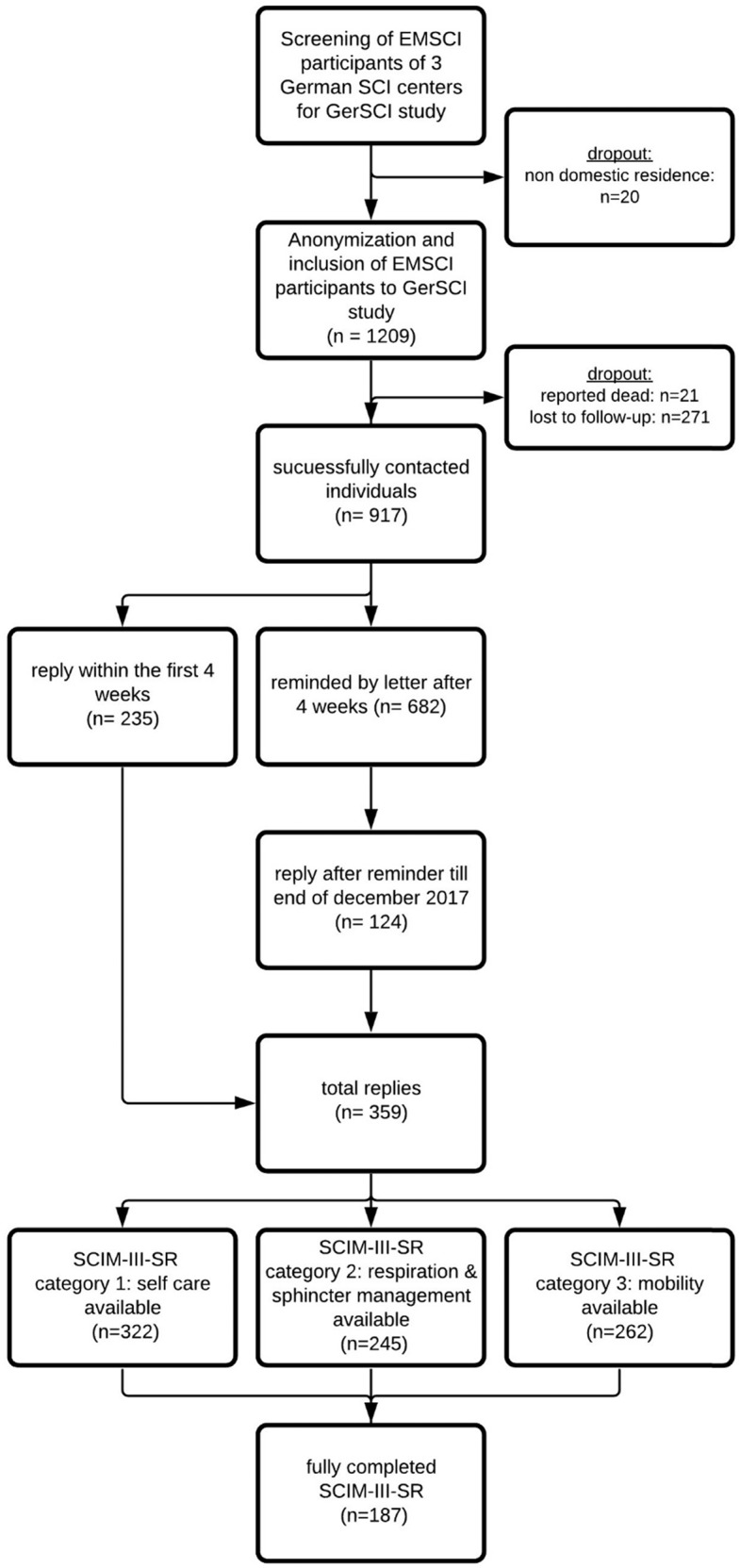
STROBE flowchart. Flowchart in line with the STROBE (Strengthening the Reporting of Observational Studies in Epidemiology) statement (http://www.strobestatement.org) illustrating the process, numbers, and dropout of study participants.

A total of 1209 individuals enrolled in the EMSCI data collection of the study centers met the GerSCI inclusion criteria. Due to a foreign residency, 20 individuals could not be included in the GerSCI survey. In 21 cases we were informed about the death of the individuals and 271 individuals could not be contacted, e.g., due to invalid address information (Fig. 1). We were able to contact 917 (75.85%) persons successfully.

Within the four weeks response time to the initial invitation for GerSCI participation, 235 replies were received. After an additional reminder, 124 additional responses were collected. Until February 2018, in total 359 questionnaires were received corresponding to 39.15% of all successfully contacted individuals.

Key demographic and neurologic lesion characteristics of responding individuals are shown in Table 1 grouped by para- and tetraplegia as last documented in the EMSCI database [13]. The majority (74.4%) of responding persons are male with a mean last observed SCIM-III total score of 61.67 (SD 26.70) points. The average total follow-up is 81.47 (SD 51.70) months. In 82.70% of all cases, the average follow-up time was longer than 24 months.

**Table 1:** Demographic and neurologic characteristics of individuals responding to the GerSCI survey.

	Classification of para- and tetraplegia according to the EMSCI database		
	Paraplegia (*n* = 170)	Tetraplegia (*n* = 189)	Total (*n* = 359)
*Age at follow-up (years)*			
Mean	53.82	58.29	56.18
Range	20–87	19–90	19–90
Median	53.00	62.00	57.00
SD	15.20	18.42	17.10
*Gender*			
Male	126 (74.1%)	141 (74.6%)	267 (74.4%)
Female	44 (25.9%)	48 (25.4%)	92 (25.6%)
*ASIA Impairment Scale according to the latest available EMSCI examination*			
A	66 (38.8%)	39 (20.6%)	105 (29.2%)
B	19 (11.2%)	15 (7.9%)	34 (9.5%)
C	25 (14.7%)	25 (13.2%)	50 (13.9%)
D	59 (34.7%)	108 (57.1%)	167 (46.5%)
Missing	1 (0.6%)	2 (1.0%)	3 (0.9%)

Demographic and neurologic characteristic of individuals who participated in the GerSCI survey. ASIA Impairment Scale definition: A = no motor or sensory function is preserved in the sacral segments S4–S5; B = sensory but not motor function is preserved below the neurological level and includes the sacral segments S4–5 (light touch or pinprick at S4–5 or deep anal pressure) AND no motor function is preserved more than three levels below the motor level on either side of the body. C = motor function is preserved at the most caudal sacral segments for voluntary anal contraction (VAC) OR the patient meets the criteria for sensory incomplete status (sensory function preserved at the most caudal sacral segments S4–5 by LT, PP, or DAP), and has some sparing of motor function more than three levels below the ipsilateral motor level on either side of the body. (This includes key or non-key muscle functions to determine motor incomplete status). For AIS C—less than half of key muscle functions below the single NLI have a muscle grade ≥ 3; D = motor function is preserved below the neurological level, and at least half of key muscles below the neurological level have a muscle grade of 3 or more.

*ASIA* American spinal injury association.

Further analyzing key demographic and lesion characteristics of all non-responding and non-successfully contacted individuals (*n* = 850) showed a mean difference of age at the point of contacting of +3.12 (mean: 59.30 vs. 56.18) years, a slightly increased proportion of female individuals of +4.40% (30.00% vs. 25.60%) and an increased time since SCI onset of +1.36 (mean: 8.78 vs. 7.42) years. The proportion of tetraplegia did only differ minor (+0.3%) from those of the responding individuals. There were no significant center effects for key demographic characteristics for responding and non-responding individuals. Only the difference in time since SCI onset was statistically significant (Mann–Whitney–U-testing: *U* = 120255.50, *p* = 0.01, *n* = 1209) with a merely weak effect size according to Cohen.

There is conformity of self-reported classification of para-/tetraplegia and the classification according to the last available ISCNSCI neurological level of injury (NLI) documented in the EMSCI database. The NLI refers to the most caudal segment with intact sensory and motor function [14]. Tetraplegia was divided into two subgroups with NLI of C1–C5 and C6–Th1 as suggested by the International Spinal Cord Society. There is good agreement of self-reported level of lesion (tetra-/paraplegia) within GerSCI to EMSCI data for all responding individuals (85.53% for NLI from C1 to C5, 79.50% for NLI C6-T1, and 83.50% for NLI rostral to Th1).

### SCIM-III-SR analysis

**Fig. 2:**
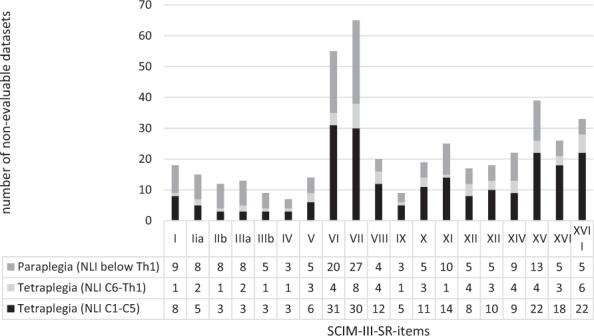
Histogram of the number of non-evaluable SCIM-III-SR items of the GerSCI questionnaires grouped by level of the lesion. Legend: I: Feeding, IIa: Bathing (upper extremities), IIb: Bathing (lower extremities), IIIa: Dressing (upper extremities), IIIb: Dressing (lower extremities), IV: Grooming, V: Respiration, VI: Sphincter management bladder, VII: Sphincter management bowel, VIII: Use of the toilet, IX: Mobility in bed, X: Bed to wheelchair transfer, XI: Wheelchair to toilet transfer, XII: Mobility I, XIII: Mobility II, XIV: Mobility III, XV: stair management, XVI: Wheelchair to car transfer, XVII: Ground to wheelchair transfer. NLI: Neurological level of the lesion.

As can be seen from Fig. 1, from all 359 questionnaires considered for further analyses 187 (52.09%) included a complete correctly filled out SCIM-III-SR section corresponding to 15.47% of all 1209 individuals eligible for the survey. In the incomplete 172 questionnaires, in total 436 items were not completely evaluable because of incoherent responses to connected items with conditional response options or missing answers. Figure 2 provides information on these items grouped by lesion level (tetra-/paraplegia) according to the EMSCI database. As displayed in Fig. 1, in total 187 SCIM-III-SR sum-scores, 322 self-care sub-scores, 245 respiration, and sphincter management sub-scores and 262 mobility sub-scores were completed and analyzed. As can be seen from Fig. 2 apart from a baseline of 10–20 non-evaluable entries per item, the items bladder management (VI), bowel management (VII), stair management (XV), transfer from wheelchair to the car (XVI), and transfer from ground to a wheelchair (XVII) contained the highest number of non-evaluable entries and were thereby identified as error-prone. Analysis showed no correlation between the education degree according to the classification of the UNESCO [12] as generated from the GerSCI questionnaire and the number of non-evaluable SCIM-III-SR items. A weak regression between the age of the individuals and the number of non-evaluable items was identified. With the increasing age of individuals also the number of non-evaluable items increases (F (1,356) = 8.561, *p* = 0.004) corresponding to a weak effect size of *f* = 0.15 according to Cohen [15]. Further focusing on the items VI, VII, XV, XVI, and XVII with the highest number of non-evaluable SCIM-III-SR entries, the last documented corresponding SCIM-III item results from the EMSCI database were analyzed. Individuals being not able to answer the corresponding SCIM-III-SR item tended to have higher SCIM-III item scores in the EMSCI examination for all error-prone items. Significance in Mann–Whitney–U-testing has been seen for the items VI (*U* = −2.316, *p* = 0.02, *n* = 359), XV (*U* = −3.669, *p* = 0.00, *n* = 359), and XVII (*U* = −2.598, *p* = 0.01, *n* = 227) corresponding to a weak to moderate effect size according to Cohen for all three analyzes.

**Fig. 3:**
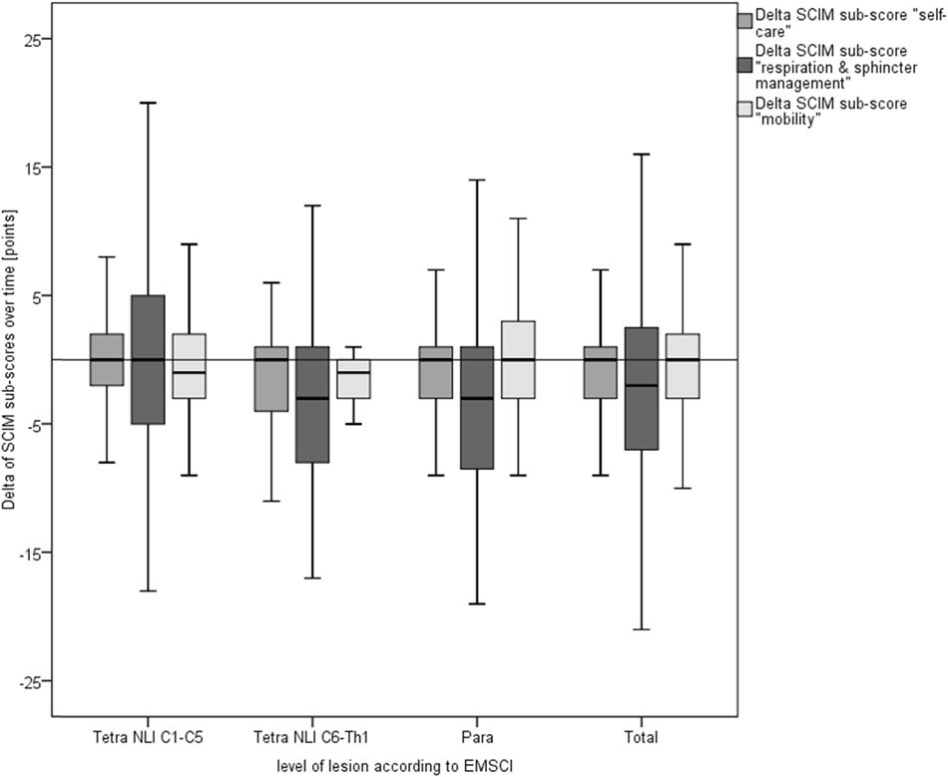
SCIM follow-up. Boxplot illustrating the delta of SCIM-III and SCIM-III-SR sub-scores over time (y-axis [points]) grouped by the level of lesion (*x*-axis) as well as cumulated for all respondents (=total). Underlying data is displayed in Table 2.

We matched the retraceable (fully completed) entries of the sub-scores (*n*_category 1_ = 322; *n*_category 2_ = 245; *n*_category3_ = 262) and the sum-score (*n* = 187) of the SCIM-III-SR from the GerSCI survey to the last SCIM-III sum-score and sub-scores of each individual in the EMSCI database. The detailed results can be found in Table 2 and Fig. 3 divided by the sub-scores and individual’s level of the lesion as documented in the EMSCI database. Due to varying numbers between followed sub-scores and sum-scores also cumulated differences of all sub-scores deviate from the difference of the sum-score over time (Table 2 + Fig. 3). The mean follow-up time was 82.54 (SD 53.28) months and thereby similar to the average follow-up of 81.47 (SD 51.70) months of all returned questionnaires. The mean last SCIM-III assessed by a clinician also closely matches one of all individuals responding (61.25 vs. 61.67 points).

**Table 2:** SCIM follow-up.

	Classification according to EMSCI database			
	Tetraplegia NLI C1–C5	Tetraplegia NLI C6–Th1	Paraplegia NLI below Th1	total
*SCIM sub-score category I—self care (0–20)*				
Sub-scores available	134	36	152	322
SCIM-III (EMSCI)	10.68	15.56	17.05	14.19
SCIM-III-SR (GerSCI)	10.31	12.92	16.16	13.36
Difference	−0.37	−2.64	−0.89	−0.83^b^
SD_difference_	4.252	5.144	3.808	4.202
*SCIM sub-score category II—respiration and sphincter management (0–40)*				
Sub-scores available	96	25	124	245
SCIM-III (EMSCI)	24.01	31.12	30.52	28.03
SCIM-III-SR (GerSCI)	23.56	26.24	27.15	25.65
Difference	−0.45	−4.88	−3.37	−2.38
SD_difference_	8.641	8.941	9.152	9.043
*SCIM sub-score category III—mobility (0–40)*				
Sub-scores available	98	29	135	262
SCIM-III (EMSCI)	15.06	21.90	20.11	18.42
SCIM-III-SR (GerSCI)	15.42	20.97	20.48	18.64
Difference	0.36	−0.93	0.37	0.22^b^
SD_difference_	7.823	9.098	6.779	7.442
*SCIM sum-score—(0–100)*				
Sum-scores available	67	21	99	187
SCIM-III (EMSCI)	48.52	69.67	68.09	61.25
SCIM-III-SR (GerSCI)	49.28	61.57	64.66	58.80
Difference^a^	0.76^a^	−8.10^a^	−3.43^a^	−2.45^a,b^
SD_difference_	18.008	21.222	14.534	16.811

SCIM follow-up divided by classification of tetraplegia/paraplegia as assigned within the EMSCI survey.

^a^Due to varying numbers between followed (fully completed) sum-scores and sub-scores also cumulated differences of all sub-scores deviate from the difference of the sum-score over time.

^b^Paired *t*-testing showed significance for differences (*p* < 0.05). Differences do not reach a weak effect size according to Cohen (*r* < 0.1).

**Fig. 4:**
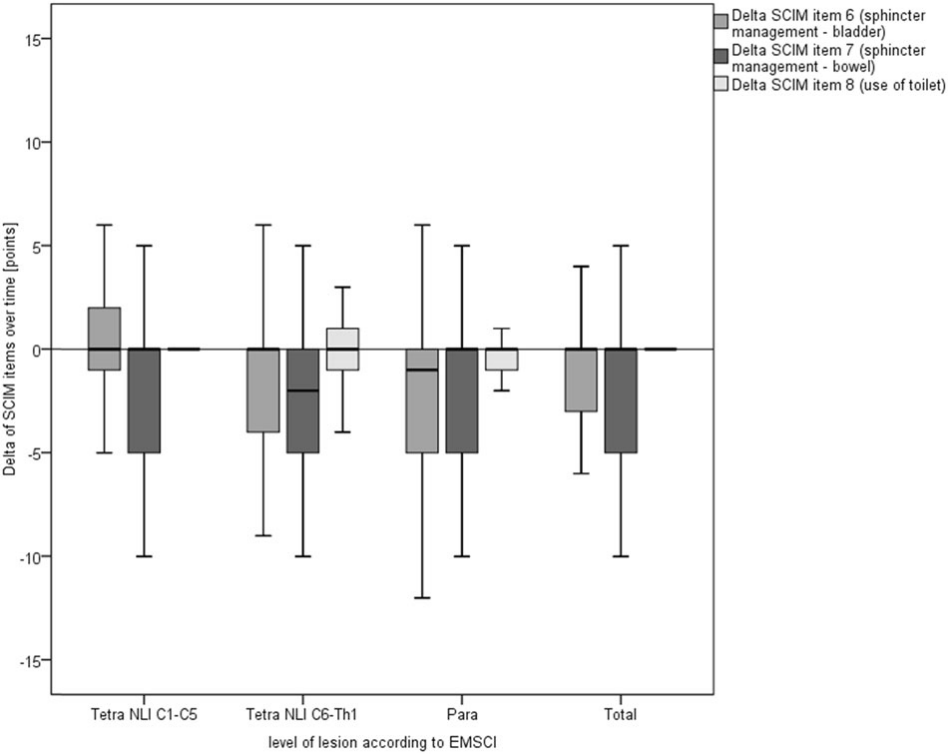
SCIM follow-up (respiration & sphincter management). Boxplot illustrating the delta of SCIM-III and SCIM-III-SR items 6–8 overtime (*y-*axis [points]) grouped by the level of lesion (*x*-axis) as well as cumulated for all respondents (=total). Numbers included: item 6: *n* = 304 (NLI C1–C5 = 119, NLI C6-T1 = 35, paraplegia = 150); item 7: *n* = 294 (NLI C1–C5 = 120, NLI C6-T1 = 31, paraplegia= 143) item 8: *n* = 339 (NLI C1–C5 = 138, NLI C6-T1 = 35, paraplegia = 166).

At first glance, SCIM-III-SR sum-score (*n* = 187) with a mean difference of −2.45 (SD 16.81) over time stayed more or less the same in comparison with the last SCIM-III assessed within EMSCI with a slight tendency of a deterioration. Further focusing on the sub-scores with a difference close to zero the sub-scores for self-care (mean difference = −0.83 points, SD 4.20) and mobility (mean difference = 0.22 points, SD 7.44) stayed stable over time but sub-score for respiration and sphincter management showed an alteration of −2.38 (SD 9.043) points. The slight deterioration of the sum score is mainly caused by this alteration. Further describing this alteration, we analyzed differences overtime for all items (items 5–8) of the sub-scale “respiration and sphincter management”. With an average change of −0.09 points (SD 0.996) the respiration score (item 5) stayed stable over time. As can be seen from Fig. 4, the deterioration is based on a lower level of independence in the bladder (mean total difference item 6: −1.20 points, SD 5.514) and bowel management (mean total difference item 7: −1.23 points, SD 4.412) over time throughout all groups. In this case too, due to varying numbers of followed items, numbers in analysis differ.

Linear regression showed no influence of the time passed since the last examination within EMSCI to the difference in SCIM-III sum- and sub-scores over time. Furthermore, no influence of time since SCI onset to SCIM-III last assessed by a clinician or SCIM differences over time was seen. Kruskal–Wallis-testing showed no center effects. Paired *t*-testing showed significant differences (*p* < 0.01) for sub-scales “self-care” and “mobility” and for sum-scores (*p* < 0.05) within GerSCI and EMSCI. These differences do not even reach a weak effect size according to Cohen (*r* < 0.1).

Focusing on the lesion level categories, also the deviation for individuals with NLI between C6 and T1 is striking (Table 2 and Fig. 3). Individuals with this lesion level tend to worsen in all sub-scores more than all other individuals. Due to the relatively small number of individuals significance only can be seen for the difference of SCIM-III sub-score addressing self-care (Mann–Whitney–U-testing: *U* = −1.994, *p* = 0.04) with a weak effect-size according to Cohen (*r* = 0.11).

### WHO-QoL and correlation with SCIM-III-SR

Individuals rated their overall QoL (WHO-QoL-BREF item 1—“How would you rate your quality of life?”) with 3.43 and their independence in an everyday setting (WHO-QoL-BREF item 17—“How satisfied are you with your ability to take care of everyday tasks?”) with 3.23 on a five-tier Likert-scale. Kruskal–Wallis-testing showed no center effects or significant differences for overall QoL between individuals with tetra- or paraplegia but a significantly higher perceived independence (Chi-Square (2) = 25.623; *p* < 0.01) for individuals with paraplegia compared to individuals with tetraplegia with NLI C1–C5 (post hoc Dunn–Bonferroni-testing: *z* = −5.048, *p* < 0.01).

The results of the Spearman-Rho correlation of the SCIM-III-SR sum-score and the sub-scores to the selected items are shown in Table 3. The numbers of individuals included in the correlation analyses vary due to the partly usable sub-scale scores of the SCIM-III-SR and the numbers of evaluable answers to the WHO-QoL-BREF items within the GerSCI questionnaires. The numbers included are shown in Table 3. A significant correlation (*p* < 0.01) of all SCIM-III-SR sub-scores and for the sum-score can be seen with moderate to partly strong effect-size (*r* > 0.5) for both selected items of WHO-QoL-BREF [15]. Detailed information on single sub-scores and target measurements selected can be found in Table 3.

**Table 3:** Correlation of SCIM-III-SR to WHO-QOL-BREF.

	WHO-QoL-BREF:	
	“How would you rate your quality of life?” (1 [very poor]–5 [very good]) (WHO-QoL-BREF-1)	“How satisfied are you with your ability to take care of everyday tasks?” (1 [very dissatisfied]–5 [very satisfied]) (WHO-QoL-BREF-17)
*SCIM-III-SR*		
*Sub-score 1*		
Correlation coefficent	*r* = 0.371^a^	*r* = 0.482^a^
*N*	304	313
*Sub-score 2*		
Correlation coefficent	*r* = 0.248^a^	*r* = 0.334^a^
*N*	235	242
*Sub-score 3*		
Correlation coefficent	*r* = 0.266^a^	*r* = 0.399^a^
*N*	247	254
*Sum-score*		
Correlation coefficent	*r* = 0.310^a^	*r* = 0.409^a^
*N*	178	185

Spearman-Rho’s correlation of SCIM-III-SR to selected target measurements of WHO-QoL-BREF (1 + 17) included in the GerSCI questionnaire. Sub-score 1 = self-care, sub-score 2 = respiration and sphincter management, sub-score 3 = mobility.

^a^Correlation is significant at level 0.01 (2-sides).

## Discussion

It can be concluded that the functional independence achieved by rehabilitative measures during primary rehabilitation was successfully maintained in the home environment after individuals’ discharge from inpatient rehabilitation. As hypothesized, we did only identify minor differences between the last SCIM-III score obtained within EMSCI and the GerSCI related SCIM-III-SR, which was determined on average 81 months later. Additionally, as initially expected we found a moderate to strong positive relationship between individuals’ functional independence and their reported QoL.

However, deterioration of functional independence related to the SCIM sub-scale of management of bowel and bladder was observed. It is questionable that the small deterioration of the respective sub-scores is linked in general to a relevant loss of independence in individuals’ daily life but we emphasize that this deterioration is linked to a worsening of management of bowel and bladder. In particular, our findings are consistent with former surveys characterizing bladder and bowel dysfunction a rising and even life-limiting functional problem for individuals with SCI over time mostly based on growing incontinency and obstipation [16–18].

Before the GerSCI study, there were no reliable, systemically collected, community-based data available about the subjective wellbeing or the life situation of individuals affected by SCI in Germany [19]. There are existing registries and databases, e.g., the EMSCI database, but they focus on clinical data. The GerSCI study gave us the unique possibility to systemically combine clinical data with an exploratory cross-sectional study [19]. Thereby our group had the opportunity to analyze individuals’ long-time course of functional independence and related QoL in addition to the original study objectives of the EMSCI and GerSCI survey.

The response rate of 39.15% (*n* = 359) can be considered as gratifying high.

For responding individuals’ the average age at the time of study participation, gender distribution and proportion of tetra- and paraplegia mostly fit those of the non-responders. Furthermore, almost exact matching in comparison to the responders of the nationwide GerSCI sample and good comparison to multiple other western European InSCI samples can be seen for this parameter [20]. The time between SCI onset and current age was slightly increased for the non-responding individuals. However, since this only shows significance with a weak effect size for the difference in time since SCI onset between responders and non-responders, we believe that the representativeness of the results from our study is comparable to other cohort studies such as the Swiss study (SwiSCI) [8].

Analyzing the returned questionnaires showed that filling in a self-reported SCIM-III questionnaire imposed a higher challenge to the participants than initially expected. In 2013, a Swiss validation study under restricted conditions (e.g., inpatient-recruitment as well as the exclusion of patients with severe health conditions and cognitive impairments) showed a quote of missing entries in 8.1% of the SCIM-III-SR assessments. We recorded an almost six times higher rate of 47.91% under unsupervised conditions [8, 21].

Particularly challenging were items concerning bladder and bowel management including connected items with conditional response options. It has to be pointed out that our data does not indicate a general trend of the inability to correctly fill out the questionnaire considering tetra- and paraplegia, complete and incomplete lesions, or the level of formal education. Solely striking in the analysis are cases connected with higher age and a higher score of the corresponding SCIM-III item at the EMSCI survey showing a significantly lower ability to correctly fill out corresponding SCIM-III-SR items. We must note, that individuals with a higher grade of independence are more challenged to complete a SCIM-III-SR questionnaire. Explicit research concerning these difficulties is lacking. We saw more frequent non-selection or faulty multiple selections of items with increasing independence. This may be due to a more difficult selection of the best-fitting item.

In this context, we strongly suggest offering a low-threshold possibility of using an online questionnaire in future surveys to avoid incoherent responses. Whenever staff resources are available, structured telephone interviews could be performed. For the future use of paper-pencil questionnaires, we suggest providing more detailed and case-related fill-in instructions at least for the error-prone items concerning bladder and bowel management.

In addition, we were able to show a moderate to the strong relationship between the SCIM-III-SR sum-score and sub-scores and our selected items for life-quality from WHO-QoL-BREF. As this instrument is supposed to be the most acceptable and established one for QoL after spinal cord injury [2] we believe that our results are in line with the common understanding of functional independence and QoL as affiliated outcome measures of initial rehabilitation investigating aspects not necessarily connected with one another [22].

Taken together, our data suggest that comprehensive primary rehabilitation efforts by dedicated SCI centers achieve levels of independence and QoL which remain stable over time. Only independence in bowel and bladder management appears to deteriorate. This requires more detailed analysis for better understanding and possible prevention.

## Limitations

This study has several limitations: according to the EMSCI inclusion and exclusion criteria only traumatic and ischemic causes were included in the survey, therefore excluding a substantial and growing portion of non-traumatic causes of SCI such as tumors or degenerative diseases.

Several individuals were reported dead, in comparison to other western European InSCI samples an elevated quote of close to 22% of individuals eligible were lost to follow-up and a substantial number of individuals did not participate.

Also following individuals by using the SCIM-III-SR is a limitation. Even though formerly validated with comparable validity to the SCIM performed by clinicians, the results might be biased by the unsupervised self-report [8]. The poor reporting for items connected to the bladder- and bowel management also might impair the reliability of our results. Nonetheless, it has to be pointed out that only a weak to moderate effect of increasing age and independence of individuals were identified as significant factors for poor reporting of SCIM-III-SR.

Furthermore, data have been collected in only 3 out of the overall 28 centers specialized in the rehabilitation of people with SCI in Germany. Since treatment facilities are quite comparable between centers in Germany, we have not seen any center effects and the demographic and lesion characteristics of our sample almost exactly match those of the whole GerSCI sample we believe that our findings are reliable enough for generalized conclusions about the long-term progression of the functional independence in a community-dwelled environment. As additional proof, the distribution of the American spinal injury impairment scale (AIS) grades in the investigated cohort also closely match the one recorded in all EMSCI centers [23]. Taken together, we strongly believe that our results concerning independence and connected QoL are representative of a German collective of individuals with traumatic or ischemic SCI and possibly might be even transferable to other health care systems of industrialized countries.

Another limitation is the restriction of our QoL analyses to only two items of the WHO-QOL-BREF questionnaire of the GerSCI survey. Life quality is an extensive, multi-faceted and somewhat contradictory concept hardly to be described in six dimensions according to the WHO-QoL-BREF approach [24]. Even multi-dimensional complex surveys like the SF-36 and the “Satisfaction with life survey” (SWLS) are accompanied by limitations and do not automatically grant a widely accepted measurement of QoL [25]. The focus on only two specific items of the WHO-QoL-BREF reduces our possibilities to investigate life quality to a one-dimensional approach. Furthermore, QoL is not solely impacted by functional independence. For example, perceived high distress or exhaustion in accomplishing related demands (e.g., self-care or sphincter management) could negatively affect individuals QoL even without any real functional deterioration.

### Strengths

The high-quality documentation of clinical data in the ISO-9001 certified EMSCI network is a clear strength of this analysis. Also, the size of the analyzed cohort renders the conclusions sound. The approach of comparing each individual’s observed SCIM score to the long-term follow-up self-reported SCIM score by data pooling is unique. The results provide new insights about the course of functional independence and limitations of a truly self-reported SCIM in home-dwelling collectives.

## Conclusion

In summary, we were able to create a long-term follow-up that demonstrates the stability of functional independence in large parts. In addition, we identified a slight deterioration concerning sphincter and bowel management in a home-dwelling environment. We were able to identify and analyze error-prone parts of the self-reported SCIM-III questionnaire, which have not been previously reported. Finally, we were able to show a moderate to the strong relationship between SCIM-III-SR scores and the perceived QoL.

We conclude that empowering individuals with the highest level of independence achievable within their primary rehabilitation is a long-lasting investment in their quality of life.

## Data Availability

The dataset generated and analyzed in the current study is available from the corresponding author on request.
